# Inability to Work Fulltime and the Association with Paid Employment One Year After the Work Disability Assessment: A Longitudinal Register-Based Cohort Study

**DOI:** 10.1007/s10926-024-10212-z

**Published:** 2024-05-31

**Authors:** Henk-Jan Boersema, Tialda Hoekstra, Raun van Ooijen, Sander K. R. van Zon, Femke I. Abma, Sandra Brouwer

**Affiliations:** 1 Department of Health Sciences, Community and Occupational Medicine, University of Groningen, University Medical Center Groningen, 9700 RB Groningen, PO-box 30001, Groningen, The Netherlands; 2 Research Center for Insurance Medicine (KCVG), Amsterdam, The Netherlands; 3 The Institute for Employee Benefit Schemes (UWV), Dutch Social Security Institute, Amsterdam, The Netherlands

**Keywords:** Work capacity evaluation, Disability evaluation, Employment, Registry data, Chronic diseases

## Abstract

**Objectives:**

Disability benefit applicants with residual work capacity are often not able to work fulltime. In Dutch work disability benefit assessments, the inability to work fulltime is an important outcome, indicating the number of hours the applicant can sustain working activities per day. This study aims to gain insight into the association between inability to work fulltime and having paid employment 1 year after the assessment.

**Methods:**

The study is a longitudinal register-based cohort study of work disability applicants who were granted a partial disability benefit (*n* = 8300). Multivariable logistic regression analyses were conducted to study the association between inability to work fulltime and having paid employment 1 year after the assessment, separately for working and non-working applicants.

**Results:**

For disability benefit applicants, whether working (31.9%) or not working (68.1%) at the time of the disability assessment, there was generally no association between inability to work fulltime and having paid employment 1 year later. However, for working applicants diagnosed with a musculoskeletal disease or cancer, inability to work fulltime was positively and negatively associated with having paid employment, respectively. For non-working applicants with a respiratory disease or with multimorbidity, inability to work fulltime was negatively associated with paid employment.

**Conclusions:**

Inability to work fulltime has limited association with paid employment 1 year after the disability benefit assessment, regardless of the working status at the time of assessment. However, within certain disease groups, inability to work fulltime can either increase or decrease the odds of having paid employment after the assessment.

**Supplementary Information:**

The online version contains supplementary material available at 10.1007/s10926-024-10212-z.

## Introduction

For people with long-term disabilities, it is often difficult to continue in fulltime jobs due to their health conditions. Reduction of working hours may accommodate these workers to continue in paid employment as working in a part-time job may better match with their residual work capacity [1]. In the Netherlands, the process to receive a disability benefit encompasses many steps. The Work and Income Act (WIA) allows employees to apply for a disability benefit after 2 years of sick leave [2]. As part of the Dutch disability benefit assessment, long-term sick-listed workers who apply for disability benefits are assessed on their (in)ability to work fulltime. The (in)ability to work fulltime is one of the key outcomes of the disability benefit assessment, and indicates the number of hours the applicant can sustain working activities per day, and is assessed by insurance physicians from the Dutch Social Security Institute: The Institute for Employee Benefits Schemes (UWV). In previous research, we found that about 40% of the workers who apply for disability benefits are assessed as being unable to work fulltime [3]. We also found a large variety between different disease groups, i.e. especially applicants with diagnoses associated with energy deficits, like diseases of the blood, have a higher likelihood of being assessed with inability to work fulltime [3]. Moreover, (in)ability to work fulltime is associated with factors like age, gender, educational level and multimorbidity. Applicants with higher age, higher educational level and multimorbidity, and women have a higher chance of being assessed with inability to work fulltime [3].

Being unable to work fulltime does not mean that these workers are not able to work at all. Most of the applicants are assessed as having residual work capacity and receive partial work disability benefit. Besides financial compensation they are also supported by the UWV to find a suitable new job or to accommodate their current job in a way that the work requirements match with their residual work capacity. From previous research, it is known that reduction of working hours may accommodate workers in the return to work process [1, 4]. Högelund and Holm [5] found that reduced working hours was the most common (about one-third) workplace accommodation among a sample of sick-listed workers. Another multinational cohort study described that working hours adaptations were significantly related to earlier sustainable return to work for applicants with chronic occupational back pain [6]. Butler et al. [7] found that workers with permanent partial impairments who returned to work with work accommodations as reduced working hours had significantly more stable labour market attachment than workers who did not have work accommodations. In a more recent survey study, it was found that about half of the employed cancer survivors received reduced hours and that receipt of this type of workplace accommodation strongly increased the continued employment of cancer survivors 5 years after diagnosis [8]. Within the Dutch social security system, it is known that having paid employment at the time of the disability assessment has major impact on labour participation in later years: those working (part-and fulltime) and having paid employment continue to participate more [9–11]. In addition, employees who continue working with their employer may inform their supervisor about their limitations. A positive experience involving the supervisor is associated with sustainable employment [12]. Employees who need to find a new job may be less likely to disclose their limitations, as in the Dutch social security setting employees are not obliged to disclose this information. Gaining insight into the impact of inability to work fulltime as an outcome of work disability assessment and having paid employment after the assessment among workers and non-workers at the time of assessment and applicants with different diagnoses may help develop approaches to support applicants assessed with an inability to work fulltime.

Within this context, the aim of this study was to examine the association between inability to work fulltime and paid employment 1 year after the disability benefit assessment in a nationwide register study of Dutch applicants who applied for disability benefits and were granted a partial disability benefit. The second aim was to study if the association is moderated by sociodemographic and disease-related factors. We conducted the analyses for applicants who had and did not have paid employment at the time of the work disability benefit assessment separately, as having paid employment at the time of the assessment is known to have an effect on labour participation in later years.

## Methods

### Setting

In the Netherlands, long-term disability benefits can be applied for under the Work and Income Act (WIA) Netherlands [2] after 2 years of sick leave by both employed and unemployed workers. The insurance physician of UWV evaluates the health situation of an applicant and first determines the residual work capacity based on several criteria. Applicants are assessed with no residual work capacity on specific conditions: (1) total work capacity loss within three months, (2) terminal disease with foreseeable total work capacity loss, (3) fluctuating work capacity, (4) hospitalization, or (5) lack of self-reliance due to severe mental or physical disorders [13]. If any of these apply, the insurance physician determines (permanent or non-permanent) full work disability. If applicants have residual work capacity, the insurance physician continues the assessment indicating potential limitations caused by their disease using the 106 items of the functional ability list (FAL) [14, 15]. The 106 items of the FAL are categorized into six domains: personal functioning (30 items, e.g. focussing attention, dividing attention), social functioning (17 items, e.g. dealing with conflicts, working with others), dynamic movements (31 items, e.g. walking, use of hand and fingers), static posture (11 items, e.g. sitting at work, standing), adjusting to environment (13 items, e.g. working in an environment with dust, smoke), and working hours (4 items, e.g. number of hours per day, working nights). One of these items involves a conclusion about the (in)ability to work fulltime, reported as the number of hours the applicant can sustain working activities per day. Particularly energy deficit, fatigue and increased need for rest are primary indicators of inability to work fulltime [16]. Following the medical disability benefit assessment by the insurance physician, a labour expert of the UWV evaluates which jobs are still considered possible with or despite the assessed limitations and the earning capacity of the applicant. The disability benefit amount is determined based on the income loss, comparing the pre-sick leave earnings in the former job with the income that can be generated in other jobs that is still considering possible with the assessed residual work capacity. A threshold exists at an income loss of 35% (for a percentage lower than 35% income loss, no benefits are granted), and a threshold of 80% income loss (to be granted a full disability benefit). A result might be that an applicant has other limitations than not being able to work fulltime, but due to these limitations cannot continue to work in his current job, but would fit a job that comes along a lower salary. This reduction of the salary can be more than 80% or between 35 and 80%. After the assessment of the limitations by the insurance physician and the evaluation of the earning capacity by the labour expert, applicants may fall into four categories: (1) full and permanent work disability (permanent > 80% income loss based on the limitations), (2) non-permanent but full work disability (> 80% income loss based on the current limitations, but prospects of improved health condition and/or limitations), (3) partial work disability (35–80% income loss based on the assessed limitations), or (4) no work disability (< 35% income loss based on the assessed limitations). Individuals in the last two groups have residual earnings capacity and are encouraged to continue (part-time) employment with their current employer or seek a new (part-time) job that aligns with their residual work capacity. For the current study, we focussed on the applicants with a partial work disability benefit (category 3). The UWV holds register data of these assessments, and has access to data on paid employment and income of all residents of the Netherlands. Within the Dutch system, employers do not have insight into the (results of the) work disability benefit assessments and the functional limitations of the applicants.

### Data

Data on sociodemographic factors, diagnoses and assessment outcomes including the estimation of inability to work fulltime were derived from the disability benefit assessment register of UWV and included all disability benefits assessments in 2016. These data were linked to register data on work status and income at the time of assessment up to 1 year after the assessment. UWV provided anonymized data. When studying the association between inability to work fulltime and having paid work, a follow-up of 1 year is suitable as it is likely that changes in the health-, social and societal situation impacting return to work will occur when the follow-up period increases. Longer follow-up makes it difficult to disentangle the impact of the inability to work fulltime assessment with impact from these changes.

### Design and Study Sample

**Fig. 1:**
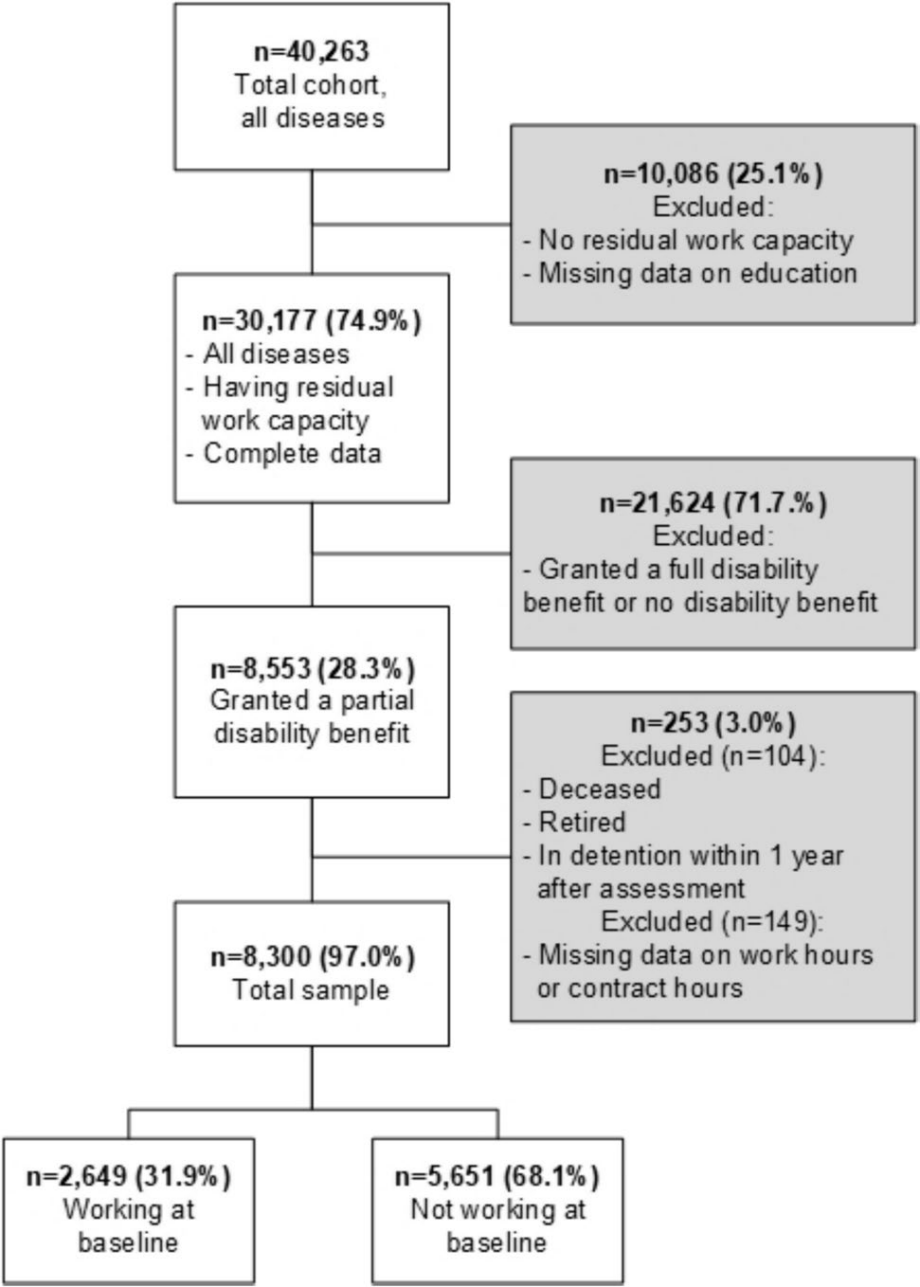
Flow chart of the study sample

The study is a longitudinal register-based cohort study of work disability applicants assessed with residual work capacity and who were granted a partial disability benefit in 2016. In 2016, *N* = 40,263 workers applied for a disability benefit. Of these, *N* = 30,177 (74.9%) were assessed with residual work capacity [2]. For this study, only applicants with residual work capacity who were granted a partial disability benefit were included. Therefore, applicants granted no (30.5%) or full (41.2%) disability benefit were excluded (*n* = 21,624, 71.7%). Additionally, applicants who died, retired, or were detained for a period of time, within 1 year after the assessment (*n* = 104), and those with missing data on the variables included in the analyses (*n* = 149) were also excluded from the study. The final study sample for the current study included *n* = 8300 applicants, which was 20.6% of all work disability benefit applicants in the Netherlands in 2016; see Fig. 1. The Medical Ethics Review Board of the University Medical Center Groningen concluded (METc 2018/570, 23-10-2018) that this study is not clinical research with human subjects as meant in the Medical Research Involving Human Subjects Act (WMO).

## Measures

### Dependent Variable: Paid Employment

Paid employment was defined as being employed and working with income of 12 h or more per week, in line with previous research [17]. Not having paid employment was defined as not working or working with income less than 12 h per week. For the determination of having paid employment at baseline (i.e. at the time of disability assessment), register data on work status at four months after the disability assessment were used, as it usually takes time before employment transitions, such as termination of a contract, are administered in the income records [18]. Applicants were considered to have paid employment at 1-year follow-up when they were employed and working with income of 12 h or more per week, for at least 3 consecutive months around the period of 12 months after the date of the disability assessment.

### Independent Variable: (In)ability to Work Fulltime

The data on (in)ability to work fulltime were retrieved from the disability benefit assessment register of UWV. The (in)ability to work fulltime was assessed by an insurance physician as part of the disability benefit assessment, for which the insurance physicians complete a 106-item Functional Ability List [14, 15]. Within the Functional Ability List, (in)ability to work fulltime is reported by the number of hours per day an applicant is able to work on a five-point scale ranging from no more than 2 hours per day to at least eight hours per day. Being able to work 8 or more hours per day was considered as having a normal ability to work, and being able to work 6 hours or less per day was considered as having an inability to work fulltime.

### Sociodemographic and Disease-Related Factors.

Sociodemographic factors included age at date of assessment, sex (male/female), educational level and working hours before sick leave. Age was classified according to working life stages: early (up to 35 years), mid (35 up to 50 years) and late (50 years and older) work life stage. Data on sex classification within the UWV system concern a binary variable. Educational level was categorized into low (primary school, lower vocational education, lower secondary school), middle (intermediate vocational education, upper secondary school) and high (upper vocational education, university). Working hours per week were classified as the number of hours an applicant worked the year before sick leave, which is roughly 2 years before the work disability assessment. Working less than 32 h per week was considered part-time work. The type of diagnosis was registered by the Insurance physician using the Dutch Classification of Occupational Health and Social Insurance (CAS), derived from the International Statistical Classification of Disease and Related Health Problems [19]. For generalizability, the primary, secondary and tertiary (when available) individual CAS-diagnoses were recoded to the 22 chapters of the ICD10. For example, diagnoses like epicondylitis, rheumatoid arthritis, arthrosis, spondylosis and scoliosis were recoded into the ICD10-Chapter ‘Diseases of the musculoskeletal system’. For the primary disease group in the multivariable logistic regression model, the variable was recoded into 15 categories, due to a small sample size in certain disease groups. We differentiated 14 ICD10-disease groups with 25 cases or more in the subsamples working or not working at baseline, and combined all other ICD10-chapters (with *n* < 25 in either the subsample working or not working at baseline) to the group ‘all other diseases’ (e.g. infectious and parasitic diseases, diseases of the skin, diseases related to pregnancy, childbirth and the puerperium, congenital malformations, factors influencing health status). Multimorbidity was defined as having one or more additional diagnoses from a different ICD10-chapter than the primary diagnosis.

## Statistical Methods

First, descriptive statistics were used to describe the baseline characteristics for the total sample, and separately for applicants working and not working at baseline. Baseline characteristics were compared using Chi^2^-tests. To give insight into paid employment and inability to work fulltime for each disease group, frequencies and percentages of (1) having paid employment at baseline (total group), (2) having paid employment 1 year after the disability assessment and (3) being assessed with inability to work fulltime (separately for applicants working and not working at baseline) are displayed per disease group.

Second, univariable and multivariable logistic regression analyses were performed to study the association of being assessed with an inability to work fulltime and having paid employment 1 year after the assessment. The univariable logistic regression analyses concern the crude model. The multivariable logistic regression analyses were conducted in three steps to gain insight into the effect of sociodemographic and disease-related factors on the association of inability to work fulltime on having paid employment. Model 1 was adjusted for work life stage, gender, educational level and contract hours at date of sick leave. Model 2 was additionally adjusted for disease groups, and Model 3 was additionally adjusted for multimorbidity.

Third, to examine if the associations of being assessed with inability to work fulltime and having paid employment 1 year after assessment were moderated by sociodemographic and disease-related factors, interaction terms were added to the final model (Model 3). The interaction effects of work life stage, gender, educational level, contract hours at date of sick leave, primary diagnosis (14 categories and ‘all other diseases’) and multimorbidity were all analysed separately. For primary diagnosis, the disease group neoplasms were considered the reference group. In case of a significant interaction concerning a variable with more than two categories, analyses were stratified by the moderator under investigation, adjusting for all other factors.

For the interaction analyses, a two-sided *p* value of < 0.10 was considered to indicate statistical significance, for all other analyses a *p* value of < 0.05 was considered to indicate statistical significance. IBM SPSS Statistics version 28 was used to perform the analyses.

## Results

### Sample Description

Of the analytic study sample (*n* = 8300), 68.1% of the applicants did not work at baseline, 54.2% were in their late work life stage and 45.8% were women (Table 1). Of all applicants, 44.9% were assessed with inability to work fulltime, but being assessed with inability to work fulltime varied greatly between different disease groups (Appendix Table I). Applicants working at time of the assessment were more often women (51.2%) and the majority was assessed with inability to work fulltime (55.7%) (Table 1). The applicants who were not working at baseline were less often women (43.2%) and the minority was assessed with inability to work fulltime (39.8%).

**Table 1:** Characteristics of the applicants, and differences between applicants working and not working at the time of work disability benefit assessment (baseline)

	Total group (*n* = 8300 *n* (%)	Working at baseline (*n* = 2649) *n* (%)	Not working at baseline (*n* = 5651) *n* (%)	*P* value
Work life stage				0.013
Early work life stage (up to 35 years)	1050 (12.7%)	304 (11.5%)	746 (13.2%)	
Mid work life stage (35 to 50 years)	2753 (33.2%)	853 (32.2%)	1900 (33.6%)	
Late work life stage (from 50 years)	4497 (54.2%)	1492 (56.3%)	3005 (53.2%)	
Female gender	3800 (45.8%)	1357 (51.2%)	2443 (43.2%)	< .001
Education level				< .001
Low	3381 (40.7%)	792 (29.9%)	2589 (45.8%)	
Middle	3023 (36.4%)	990 (37.4%)	2033 (36.0%)	
High	1896 (22.8%)	867 (32.7%)	1029 (18.3%)	
Contract hours at date of sick leave (> 32 h per week)	5812 (70.0%)	1797 (67.8%)	4015 (71.0%)	0.002
Inability to work fulltime	3723 (44.9%)	1476 (55.7%)	2247 (39.8%)	< .001
Having paid employment at one year follow-up	2872 (34.6%)	2204 (83.2%)	668 (11.8%)	0.000
ICD10 Disease groups				
Neoplasms	566 (6.8%)	304 (11.5%)	262 (4.6%)	< .001
Diseases of the blood and blood-forming organs	108 (1.3%)	44 (1.7%)	64 (1.1%)	
Endocrine, nutritional and metabolic disorders	127 (1.5%)	37 (1.4%)	90 (1.6%)	
Mental and behavioural disorders	3062 (36.9%)	797 (30.1%)	2265 (40.1%)	
Diseases of the nervous system	329 (4.0%)	158 (6.0%)	171 (3.0%)	
Diseases of the eye and adnexa	61 (0.7%)	29 (1.1%)	32 (0.6%)	
Diseases of the ear and mastoid process	80 (1.0%)	25 (0.9%)	55 (1.0%)	
Diseases of the circulatory system	626 (7.5%)	267 (10.1%)	359 (6.4%)	
Diseases of the respiratory system	178 (2.1%)	56 (2.1%)	122 (2.6%)	
Diseases of the digestive system	153 (1.9%)	59 (2.2%)	94 (1.7%)	
Diseases of the musculoskeletal system	1940 (23.4%)	509 (19.2%)	1431 (25.3%)	
Diseases of the genitourinary system	94 (1.1%)	49 (1.8%)	45 (0.8%)	
Symptoms, signs and abnormal clinical and laboratory findings	382 (4.6%)	97 (3.7%)	285 (5.0%)	
Injury, poisoning and other consequences of external causes	474 (5.7%)	171 (6.5%)	303 (5.4%)	
All other diseases	120 (1.4%)	47 (1.8%)	73 (1.3%)	
Multimorbidity	4256 (51.3)	1219 (46.0%)	3037 (53.7%)	< .001

### Associations of Inability to Work Fulltime and Paid Employment

For applicants working at baseline, being assessed with inability to work fulltime was significantly associated (OR 1.31, 95%CI 1.07–1.61) with having paid employment 1 year after the assessment in the crude model (Table 2). The association remained significant after adjusting for sociodemographic factors (OR 1.32, 95%CI 1.07–1.62) but not after additional adjustment for disease-related factors (OR 1.15, 95%CI 0.92–1.43). For applicants not working at baseline, no significant associations between the assessment of inability to work fulltime and having paid employment 1 year after the assessment were found.

**Table 2:** Associations of inability to work fulltime and having paid employment 1 year after the assessment, stratified by working and not working at baseline (univariable and multivariable logistic regression analyses)

	Working at baseline (*n* = 2649)			Not working at baseline (*n* = 5651)		
	OR	95% CI	*p* value	OR	95% CI	*p* value
Univariable	1.312	1.070–1.609	.009	1.061	0.900–1.250	.480
Multivariable						
Model 1*	1.316	1.070–1.620	.009	0.958	0.804–1.141	.631
Model 2**	1.143	0.919–1.422	.229	0.942	0.783–1.133	.525
Model 3***	1.148	0.923–1.428	.215	0.935	0.777–1.125	.476

*OR* Odds ratio, *CI* Confidence interval

*Model 1: Adjusted for work life stage, gender, educational level, contract hours at date of sick leave

**Model 2: Adjusted for all variables of Model 1 and for disease groups

***Model 3: Adjusted for all variables of Model 2 and for multimorbidity

### Moderation of Inability to Work Fulltime by Sociodemographic and Disease-Related Factors

Inability to work fulltime was not significantly moderated by sociodemographic factors in both the working and non-working applicants. For the applicants working at baseline, the interaction of inability to work fulltime with disease groups showed a significant association with having paid employment 1 year after assessment. Similarly, for applicants who were non-working, the interaction of inability to work fulltime with disease groups showed a significant association with having paid employment 1 year after the assessment. Additionally, within this group, the interaction of inability to work fulltime with multimorbidity also showed a significant association (OR 0.71, 95%CI 0.51–1.00) with having paid employment 1 year after the assessment (Appendix Table II). As inability to work fulltime was significantly moderated by disease groups (a variable with more than two categories) for both the applicants working and not working at baseline, multivariable logistic regression analyses stratified to disease groups were conducted.

For applicants working at baseline and having a disease of the musculoskeletal system, being assessed with inability to work fulltime showed increased odds (OR 2.19, 95%CI 1.20–4.00) for having paid employment 1 year after the assessment. Furthermore, for applicants assessed with neoplasms, being assessed with inability to work fulltime was significant at the level of *p* < 0.10 to having decreased odds for having paid employment 1 year after the assessment (OR 0.40, 95%CI 0.13–1.19).

For applicants not working at baseline, within none of the disease groups, inability to work fulltime was significantly associated with having paid employment 1 year later. However, inability to work fulltime showed a significant association at the *p*-level < 0.10 to having decreased odds of having paid employment within the group diseases of the respiratory system (OR 0.15, 95%CI 0.02–1.12). See Table 3 for more details.

**Table 3:** Paid employment 1 year after assessment and associations of inability to work fulltime with having paid employment 1 year after the assessment, stratified by ICD10-disease groups and to working and not working at baseline (multivariable logistic regression adjusted for work life stage, gender, educational level, multimorbidity and contract hours at date of sick leave)

	Working at baseline				Not working at baseline			
ICD10 Disease groups	Paid employment 1-year follow-up *N* (%)*	OR	95% CI	*p* value	Paid employment 1-year follow-up *N* (%)*	OR	95%CI	*p* value
Neoplasms	274 (90.1%)	0.398	0.133–1.190	.099	26 (9.9%)	0.656	0.278–1.544	.334
Diseases of the blood and blood-forming organs	43 (97.7%)	0.183	0.000	1.000	14 (21.9%)	1.008	0.238–4.278	.991
Endocrine, nutritional and metabolic disorders	28 (75.7%)	0.465	0.075–2.903	.413	12 (13.3%)	0.000	0.000	.998
Mental and behavioural disorders	600 (75.3%)	1.058	0.756–1.481	.741	298 (13.2%)	1.082	0.832–1.409	.556
Diseases of the nervous system	139 (88.0%)	2.329	0.748–7.250	.144	22 (12.9%)	1.427	0.464–4.383	.535
Diseases of the eye and adnexa	28 (96.6%)	0.004	0.000	1.000	4 (12.5%)	2.090	0.037–117.336	.720
Diseases of the ear and mastoid process	19 (76.0%)	0.206	0.006–7.307	.386	0 (0%)	–**	–	–
Diseases of the circulatory system	235 (88.0%)	1.323	0.573–3.054	.512	32 (8.9%)	0.998	0.461–2.159	.996
Diseases of the respiratory system	44 (78.6%)	1.695	0.339–8.482	.521	8 (6.6%)	0.149	0.020–1.120	.064
Diseases of the digestive system	55 (93.2%)	3.259	0.088–120.880	.522	17 (18.1%)	1.819	0.403–8.209	.437
Diseases of the musculoskeletal system	433 (85.1%)	2.194	1.204–4.000	.010	148 (10.3%)	0.839	0.490–1.435	.521
Diseases of the genitourinary system	42 (85.7%)	0.132	0.009–1.966	.142	7 (15.6%)	0.474	0.057–3.930	.489
Symptoms, signs and abnormal clinical and laboratory findings	87 (89.7%)	2.464	0.468–12.967	.287	31 (10.9%)	0.562	0.216–1.461	.237
Injury, poisoning and other consequences of external causes	137 (80.1%)	0.914	0.402–2.080	.831	41 (13.5%)	0.813	0.359–1.839	.619
All other diseases	40 (85.1%)	7.536	0.244–232.292	.248	8 (11.0%)	0.434	0.035–5.382	.516

*OR* Odds ratio, *CI* Confidence interval

*N (%) within applicants working or not working at baseline

**None of the applicants were having paid employment one year after the assessment, therefore analyses were not possible

## Discussion

Our aim was to examine the association of being assessed with inability to work fulltime with having paid employment 1 year after the disability assessment, separately for applicants working and not working at the time of assessment. Our results showed that for the total sample, inability to work fulltime was not associated with having paid employment 1 year later when adjusted for sociodemographic and disease-related factors. However, our results showed that the type of chronic disease moderated the associations between the inability to work fulltime and paid employment. The inability to work fulltime increased the odds of having paid employment 1 year later for those working at baseline with musculoskeletal diseases. For those working at baseline with a neoplasm, and those not working at baseline with a disease of the respiratory system, inability to work fulltime decreased the odds of having paid employment 1 year later. Sociodemographic factors did not moderate the association between the inability to work fulltime and employment 1 year later.

In our study population, 44.9% of the applicants who were granted a partial disability 2 years after sick leave were assessed with inability to work fulltime. We did not find evidence that being assessed with inability to work fulltime supports or hinders workers with residual work capacity to remain or re-enter in paid employment in the total samples, but did find associations within specific disease groups. We found a beneficial effect of inability to work fulltime on having paid employment 1 year later for those applicants who worked at the time of assessment and were diagnosed with a musculoskeletal disease. Moreover, we found borderline significant associations (*p* < 0.10) between inability to work fulltime and paid employment for working applicants with neoplasm and for non-working applicants diagnosed with a disease of the respiratory system.

A potential explanation for the increased odds of having paid employment 1 year subsequent to being assessed with inability to work fulltime, among those working at baseline and being diagnosed with a musculoskeletal disease, could be attributed to the relatively stable prognosis of diseases of the musculoskeletal system, regarding the functional limitations. Occupational physicians and employers commonly possess an adept understanding of adjusting work to these limitations [18, 20–22]. Consequently, applicants who still (partly) work at the time of assessment stand a favourable chance of retaining adjusted work arrangements compared to those with other chronic diseases [23–25]. On the other hand, for cancer survivors, it might be difficult to stay in paid employment due to the impact on energy levels and cognitive functioning [26–28]. This may explain the negative association which was found between inability to work and paid employment 1 year after the disability assessment. The finding, for applicants not working at baseline with diseases of the respiratory system (e.g. pneumonia, emphysema, chronic obstructive pulmonary disease and pneumoconiosis), that inability to work fulltime lowers the chance of having paid work 1 year later is in line with an international survey in 2011, showing that 40% of the working population had retired prematurely because of COPD [29]. Most of the diseases of the respiratory system are chronic with a negative effect on energy and endurance and with a higher risk [3] to be unable to work fulltime. The findings on multimorbidity may also be due to be more at risk for involuntary labour market exit in comparison to those workers without or with one chronic health condition [30, 31]. Especially those applicants with multiple chronic diseases may not be able to work fulltime and will have more problems finding paid employment as they have more severe medical problems, resulting in more work limitations, than those diagnosed with one disease or those who are able to work fulltime.

### Strengths and Limitations

A strength of the study is the large sample size, including register data of a Dutch year cohort of applicants for work disability benefit, granted a partial disability benefit in 2016. Data included socio-demographics, all diagnoses and monthly work status in 2016 and 2017. Furthermore, all assessments were carried out by skilled professionals, adhering to professional guidelines and assessment methods. Although the sample size of our study is large and the data are rich, possible confounders such as severity of diseases, symptoms of the diseases and the course of the disease after the assessment, and personal (e.g. motivation) factors, environmental (e.g. support at the job) factors, and (adjusted) work accommodations that might facilitate employment, are not included in the data, which is a limitation of our study. Furthermore, having an insufficient amount of energy for work, expressed in an inability to work fulltime, also impacts other daily activities as self-care, social activities, and household and leisure activities. People with inability to work fulltime could, based on their situation, possibilities and preferences, decide or being forced to restrict working activities or restrict their activities in other fields of daily participation to stay in a certain balance [32]. We have no insight into the factors playing a role in this decision-making process from our register data. And as a final limitation, there is the probability of false-positive finding due multiple testing [33].

### Implications for Practice and Future Research

This study shows that for both working and non-working disability benefit applicants, the association of inability to work fulltime with having paid employment 1 year after the assessment is limited, but within specific disease groups, inability to work fulltime was either positively or negatively associated with having paid employment. Especially applicants with a disease of the musculoskeletal system may benefit from being assessed with inability to work fulltime. Conversely, applicants with neoplasm, a disease of the respiratory system and applicants diagnosed with more than one chronic disease, inability to work fulltime hinders having paid employment after the disability benefit assessment. Occupational and insurance physicians can integrate this knowledge in supporting workers on sick leave with return to work, and in the assessment of work disability. However, further research is needed to gain more insight into how being assessed with inability to work fulltime contributes or hinders stay at work and return to work after the disability benefit assessment.

Additionally, in our study, we only focussed on the applicants who were granted a partial disability benefit, as they are partially compensated and are expected to find a job for the part of income loss. Future research on inability to work fulltime and paid employment might include other samples as the associations of being assessed with inability to work fulltime and paid employment after the assessment might be different for those applicants who were granted a full or no work disability benefit. Furthermore, we looked at the overall effect of the assessment of inability to work fulltime, not differentiating between the number of hours an applicant is able to work. Future research could differentiate between the assessed number of hours someone is able to work per day, for example 6 or 4 hours, and whether there is a difference in paid employment 1 year after the assessment. Subsequently, we only looked into having paid employment after the assessment. To gain more detailed knowledge about the impact of the assessment of work disability on labour market participation, future research could focus on other outcomes such as the increase or decrease in number of hours someone works 1 year after the assessment.

## Conclusion

This study shows that for both working and non-working disability benefit applicants, the association of inability to work fulltime with having paid employment 1 year after the assessment is limited. However, within specific disease groups, inability to work fulltime increases or decreases the odds of having paid employment. For applicants working at baseline and diagnosed with a musculoskeletal disease, inability to work fulltime was positively associated with having paid employment 1 year later. For applicants with cancer, respiratory diseases and multimorbidity, inability to work fulltime showed a trend towards significance in being negatively associated with having paid employment a year later.

## Supplementary Information

Below is the link to the electronic supplementary material.

Supplementary file1 (DOCX 51 kb)

## Data Availability

The data that support the findings of this study were made available from UWV. However, restrictions apply to the availability of these data, which were used under licence for the current study, and are not publicly available. Data are however available from the authors upon reasonable request and with permission of UWV.
