# Supplementary material for: Inability to Work Fulltime and the Association with Paid Employment One Year After the Work Disability Assessment: A Longitudinal Register-Based Cohort Study
Source: J Occup Rehabil. 2024 May 31;35(2):390–9. doi: 10.1007/s10926-024-10212-z (PMC12089174; doi:10.1007/s10926-024-10212-z)
Supplement: Supplementary file 1 — Supplementary file1 (DOCX 51 kb) [file 10926_2024_10212_MOESM1_ESM.docx]

**APPENDIX**

Table I. Prevalence of inability to work fulltime per ICD10 disease group for the total sample and separately for working and not working at baseline.

|  | Total | | | Working at baseline | | Not working at baseline | |
| --- | --- | --- | --- | --- | --- | --- | --- |
| ICD10 Disease groups | N (%)* | Working at baseline  N (%)** | Not working at baseline  N (%)** | Inability to work fulltime  N (%)** | Ability to work fulltime  N (%)** | Inability to work fulltime  N (%)** | Ability to work fulltime  N (%)** |
| Neoplasms | 566 (6.8%) | 304 (53.7%) | 262 (46.3%) | 275 (90.5%) | 29 (9.5%) | 180 (68.7%) | 82 (31.3%) |
| Diseases of the blood and blood-forming organs | 108 (1.3%) | 44 (40.7%) | 64 (59.3%) | 40 (90.9%) | 4 (9.1%) | 55 (85.9%) | 9 (14.1%) |
| Endocrine, nutritional and metabolic disorders | 127 (1.5%) | 37 (29.1%) | 90 (70.9%) | 27 (73.0%) | 10 (27.0%) | 38 (42.2%) | 52 (57.8%) |
| Mental and behavioural disorders | 3062 (36.9%) | 797 (26.0%) | 2265 (74.0%) | 518 (65.0%) | 279 (35.0%) | 1395 (61.6%) | 870 (38.4%) |
| Diseases of the nervous system | 329 (4.0%) | 158 (48.0%) | 171 (52.0%) | 137 (86.7%) | 21 (13.3%) | 106 (62.0%) | 65 (38.0%) |
| Diseases of the eye and adnexa | 61 (0.7%) | 29 (47.5%) | 32 (52.5%) | 20 (69.0%) | 9 (31.0%) | 9 (28.1%) | 23 (71.9%) |
| Diseases of the ear and mastoid process | 80 (1.0%) | 25 (31.3%) | 55 (68.8%) | 16 (60.0%) | 9 (40.0%) | 26 (45.5%) | 29 (54.5%) |
| Diseases of the circulatory system | 626 (7.5%) | 267 (42.7%) | 359 (57.3%) | 210 (78.7%) | 57 (21.3%) | 185 (51.5%) | 174 (48.5%) |
| Diseases of the respiratory system | 178 (2.1%) | 56 (31.5%) | 122 (68.5%) | 40 (71.4%) | 16 (28.6%) | 78 (63.9%) | 44 (36.1%) |
| Diseases of the digestive system | 153 (1.9%) | 59 (38.6%) | 94 (61.4%) | 54 (91.5%) | 5 (8.5%) | 67 (71.3%) | 27 (28.7%) |
| Diseases of the musculoskeletal system | 1940 (23.4%) | 509 (26.2%) | 1431 (73.8%) | 225 (44.2%) | 284 (55.8%) | 280 (19.6%) | 1151 (80.4%) |
| Diseases of the genitourinary system | 94 (1.1%) | 49 (52.1%) | 45 (47.9%) | 44 (89.8%) | 5 (10.2%) | 36 (80.0%) | 9 (20.0%) |
| Symptoms, signs and abnormal clinical and laboratory findings | 382 (4.6%) | 97 (25.4%) | 285 (74.6%) | 62 (63.9%) | 35 (36.1%) | 110 (38.6%) | 175 (62.4%) |
| Injury, poisoning and other consequences of external causes | 474 (5.7%) | 171 (36.1%) | 303 (63.9%) | 86 (50.3%) | 85 (49.7%) | 104 (34.3%) | 199 (65.7%) |
| All other diseases | 120 (1.4%) | 47 (39.2%) | 73 (60.8%) | 35 (74.5%) | 12 (25.5%) | 33 (45.2%) | 40 (54.8%) |

* Percentage of diseases within of total sample ** Percentage of (not) working and (in)ability to work fulltime within disease groups

Table II. Interactions of inability to work fulltime with sociodemographic and disease-related factors on the association with having paid employment one year after the assessment, separately for applicants working and not working at baseline (multivariable logistic regression analysis).

|  | Working at baseline (n=2649) | | | Not working at baseline (n=5651) | | |
| --- | --- | --- | --- | --- | --- | --- |
|  | OR | 95% CI | p-value | OR | 95%CI | p-value |
| Work life stage (early – ref)*inability to work fulltime |  |  |  |  |  |  |
| Mid work life stage (35.00 to 50.00 years) | 0.693 | 0.325-1.477 | .342 | 0.759 | 0.472-1.221 | .256 |
| Late work life stage (from 50.00 years) | 1.031 | 0.499-2.127 | .935 | 0.703 | 0.433-1.142 | .155 |
| Female gender*inability to work fulltime | 0.828 | 0.541-1.266 | .383 | 1.077 | 0.762-1.523 | .675 |
| Educational level (low – ref)*inability to work fulltime |  |  |  |  |  |  |
| Mid | 0.700 | 0.407-1.203 | .196 | 1.082 | 0.737-1.587 | .688 |
| High | 0.742 | 0.435-1.264 | .272 | 1.189 | 0.756-1.872 | .454 |
| Contract hours (>32h)*inability to work fulltime | 0.928 | 0.600-1.434 | .735 | 1.193 | 0.798-1.784 | .389 |
| ICD10 Disease groups*inability to work fulltime |  |  |  |  |  |  |
| Neoplasms – reference group |  |  |  |  |  |  |
| Diseases of the blood and blood-forming organs | .000 | 0.000 | .999 | 1.481 | 0.307-7.148 | .625 |
| Endocrine, nutritional and metabolic disorders | 1.191 | 0.175-8.131 | .858 | 0.000 | 0.000 | .998 |
| Mental and behavioural disorders | 2.521 | 0.809-7.860 | .111 | 1.682 | 0.711-3.979 | .237 |
| Diseases of the nervous system | 3.958 | 0.893-17.541 | .070 | 3.000 | 0.820-10.970 | .097 |
| Diseases of the eye and adnexa | .000 | .000 | .999 | 3.911 | 0.373-41.048 | .256 |
| Diseases of the ear and mastoid process | 3.744 | 0.406-34.530 | .244 | 1.626 | 0.000 | 1.000 |
| Diseases of the circulatory system | 2.813 | 0.727-10.887 | .134 | 1.459 | 0.485-4.384 | .501 |
| Diseases of the respiratory system | 2.350 | 0.412-13.420 | .336 | 0.488 | 0.089-2.669 | .408 |
| Diseases of the digestive system | 5.063 | 0.363-70.557 | .228 | 3.743 | 0.775-18.065 | .100 |
| Diseases of the musculoskeletal system | 5.357 | 1.567-18.310 | .007 | 1.171 | 0.445-3.086 | .749 |
| Diseases of the genitourinary system | 0.810 | 0.069-9.580 | .867 | 0.755 | 0.116-4.931 | .769 |
| Symptoms, signs and abnormal clinical and laboratory findings | 3.474 | 0.619-19.504 | .157 | 0.691 | 0.199-2.400 | .561 |
| Injury, poisoning and other consequences of external causes | 2.438 | 0.645-9.212 | .189 | 1.236 | 0.406-3.769 | .709 |
| All other diseases | 1.728 | 0.215-13.856 | .607 | 0.272 | 0.027-2.742 | .269 |
| Multimorbidity*inability to work fulltime | 1.057 | 0.696-1.604 | .794 | 0.713 | 0.508-1.001 | .051 |

*OR* odds ratio*, CI* confidence interval*, ref* reference group
